# Isolation of a genetically accessible thermophilic xylan degrading bacterium from compost

**DOI:** 10.1186/s13068-016-0618-7

**Published:** 2016-10-06

**Authors:** Martinus J. A. Daas, Antonius H. P. van de Weijer, Willem M. de Vos, John van der Oost, Richard van Kranenburg

**Affiliations:** 1 Laboratory of Microbiology, Wageningen University, Stippeneng 4, 6708 WE Wageningen, The Netherlands; 2 Corbion, Arkselsedijk 46, 4206 AC Gorinchem, The Netherlands

**Keywords:** *Geobacillus*, Thermophile, Compost, Xylan, CMC, Lactic acid, Electroporation, Fermentation

## Abstract

**Background:**

Due to the finite nature of global oil resources we are now faced with the challenge of finding renewable resources to produce fuels and chemicals in the future. Lactic acid has great potential as a precursor for the production of bioplastics alternatives to conventional plastics. Efficient lactic acid fermentation from non-food lignocellulosic substrates requires pretreatment and saccharification to generate fermentable sugars. A fermentation process that requires little to no enzyme additions, i.e. consolidated bioprocessing would be preferred and requires lactic acid-producing organisms that have cellulolytic and/or hemicellulolytic activity.

**Results:**

To obtain candidate production strains we have enriched and isolated facultative anaerobic (hemi) cellulolytic bacterial strains from compost samples. By selecting for growth on both cellulose and xylan, 94 *Geobacillus* strains were isolated. Subsequent screening for lactic acid production was carried out from C6 and C5 sugar fermentations and a selection of the best lactic acid producers was made. The denitrifying *Geobacillus thermodenitrificans* T12 was selected for further research and was rendered genetically accessible. In fermentations on a mixture of glucose and xylose, a total of 20.3 g of lactic acid was produced with a yield of 0.94 g product/g sugar consumed. In addition, strain T12 is capable of direct conversion of beech wood xylan to mainly lactic acid in minimal media.

**Conclusions:**

We have demonstrated that *G. thermodenitrificans* T12 is genetically accessible and produces lactic acid as its main fermentation product on glucose, xylose and a mixture thereof. Strain T12 was additionally used for the direct conversion of xylan to lactic acid. The genetic accessibility of the T12 strain provides a solid basis for the development of this strain into a host for consolidated bioprocessing of biomass to lactic acid.

**Electronic supplementary material:**

The online version of this article (doi:10.1186/s13068-016-0618-7) contains supplementary material, which is available to authorized users.

## Background

The increasing consciousness regarding the sustainability of our current life standards has led to the accelerating development of alternative production strategies for fuels, energy and chemicals [1]. Lactic acid is an organic acid that can be used as building block for poly lactic acid (PLA) [2]. Petrochemically produced lactic acid always yields a racemic mixture of both d- and l-lactic acid which results in a lower thermostability of the PLA polymer in comparison to its optically pure counterpart derived from microbial production. Microbial production of lactic acid currently dominates the chemical synthesis alternative; however, the efficiency largely depends on the substrate, microbe and mode of production. In recent years the use of lignocellulosic or non-edible biomass as a resource has gained much interest as it does not compete with the food and feed industry. However, use of this type of substrate requires costly saccharolytic enzymes for successful conversion to fermentable sugars [3]. Consolidated bioprocessing (CBP) is a method designed to exclude such enzymes and thus optimise lignocellulosic conversion in the most economically feasible way. It is believed that this mode of integration can result in a process four times more cost-efficient even when compared to simultaneous saccharification and fermentation [4].

In the last decades substantial efforts have been made to isolate and engineer organisms for the conversion of lignocellulosic substrates. The search for suitable saccharolytic organisms even from the most extreme and remote spots on earth has led to the discovery of many novel isolates [5, 6]. Among these are isolates from the genus *Geobacillus*, a versatile group of thermophilic facultative anaerobic bacteria [7]. Geobacillus are Gram-positive, rod-shaped bacteria with the ability to sporulate. In recent years, this genus has gained much interest not only because of their thermostable enzymes, but also because of their ability to ferment C6 and C5 sugars simultaneously. Furthermore, several *Geobacillus* strains are capable of degrading xylan or cellulose [8, 9]. Xylanolytic activity is common in this genus and the responsible hemicellulose utilisation (HUS) locus, containing the majority of genes involved in the hemicellulose metabolism, has been described in detail elsewhere [10]. Cellulolytic activity has been demonstrated only in a few studies [11–13]. In addition, *Geobacillus* strains have been proven to be useful for heterologous expression of a variety of proteins [14, 15]. Nevertheless, only limited information on this genus is available, with only 18 complete genome sequences in the NCBI database and only a few strains that have been proven to be readily transformable [16–18]. Moreover, genetic accessibility of (geo) bacilli seems to be strain-specific [16, 17]. Because the lack of knowledge of the *Geobacillus* species is considered to be one of the main drawbacks to fully exploit members of this genus as platform organisms, more genome sequences, physiological data and improved genetic tools are required.

In this paper, we describe the isolation of novel *Geobacillus* strains of various species with the ability to degrade cellulose and/or xylan. The optimisation of transformation and the heterologous expression of a reporter gene are shown for one selected strain. In addition, the production of lactic acid by this strain was demonstrated on glucose, xylose and on beech wood xylan.

## Methods

### Media and cultivation methods

Cellulolytic thermophile vitamin medium (CTVM; based on [14, 17, 19, 20]) contained per L: 8.37 g MOPS and salts mix consisting of 1 g NH_4_Cl; 3 g NaCl; 1.50 g Na_2_SO_4_; 0.08 g NaHCO_3_; 1 g KCl; 1.8 g MgCl_2_ × 6H_2_O; 0.30 g CaCl_2_ × 2H_2_O. pH was set to 6.6 at room temperature and the medium was autoclaved for 20 min at 121 °C, after which 1 mL of K_2_HPO_4_ (250 g/L; pH 6.6), 10 mL of filter sterile 100× metal mix and 1 mL of filter sterile 1000× vitamin solution were added. 100× metal mix contained per liter: 1.60 g MnCl_2_ × 6H_2_O; 0.1 g ZnSO_4_; 0.2 g H_3_BO_3_; 0.01 g CuSO_4_ × 5H_2_O; 0.01 g Na_2_MoO_4_ × 2H_2_O; 0.1 g CoCl_2_ × 6H_2_O; 0.7 g FeSO_4_ × 7H_2_O; 5 g CaCl_2_ × 2H_2_O; 20 g MgCl_2_ × 6H_2_O. 1000× vitamin mix contained per L: 0.1 g thiamine; 0.1 g riboflavin; 0.5 g nicotinic acid; 0.1 g panthothenic acid; 0.5 g pyridoxamine, HCl; 0.5 g pyridoxal, HCl; 0.1 g d-biotin; 0.1 g folic acid; 0.1 g *p*-aminobenzoic acid; 0.1 g cobalamin.

LB2 contains per liter: 10 g tryptone (Oxoid), 5 g yeast extract (Roth), 10 g sodium chloride and salts mix consisting of 1 g NH_4_Cl; 3 g NaCl; 1.50 g Na_2_SO_4_; 0.08 g NaHCO_3_; 1 g KCl; 1.8 g MgCl_2_ × 6H_2_O; 0.30 g CaCl_2_ × 2H_2_O. pH was set to 6.6 at room temperature and the medium was autoclaved for 20 min at 121 °C, after which 10 mL of K_2_HPO_4_ (250 g/L) was added.

Minimal Media (MM) contained per liter: 0.52 g K_2_HPO_4_; 0.23 g KH_2_PO_4_; 0.5 g NH_4_NO_3_ (MMy) or 0.3 g NH_4_Cl (MMy+). After autoclaving, 1 mL of the following 1000× concentrated sterile stocks were added: Nitrilotriacetic acid (200 g/L); MgSO_4_ × 7H_2_O (145.44 g/L); CaCl_2_ × 2H_2_O (133.78 g/L); FeSO_4_ × 7H_2_O (11.12 g/L).

For CTVMy/MMy medium, 0.5 g/L yeast extract (Roth) was added to the medium and CTVMy+/MMy+ contains 5 g/L yeast extract (Roth).

Glycerol stocks of cultures were made by adding 500 µL of sterilised 60 % glycerol to 1.5 mL culture, in a 2 mL cryogenic vial (Corning). Stocks were stored at −80 °C.

In all plate and tube cultures, carbon substrates were used in a concentration of 10 g/L unless stated otherwise. Carbon sources were autoclaved separately with xylose being filter sterilised.

For plate cultures, 5 g/L gelrite (Roth) was added. Anaerobic cultivation of plates was done in an anaerobic jar (HP0011, Oxoid) containing an AnaeroGen sachet (AN0035, Oxoid).

All wild-type strains were isolated and cultured at 65 °C. Strains harbouring the pNW33n plasmid are always cultured at 55 °C to maintain the plasmids replication. Cultures in liquid media are shaken at 150 RPM unless stated otherwise.

### Sampling

Samples were collected from both a mature and an active compost heap at ReCom Ede (NL). The temperature of the mature compost was around 35 °C at the sampling site on top of the compost heap and around 65 °C for the active compost heap which was sampled at a depth of 30 cm. Both heaps were semi-anaerobic due to mixing once every week. The sampling site of the 1st isolation was aerobic since the sampling was done on top of the compost. Samples were taken by scraping compost into a plastic jar and were used to inoculate immediately after transport to the lab at room temperature. The sample of the 2nd isolation was kept under anaerobic conditions during transport using an Oxoid AnaeroGen sachet in a sealed anaerobic box.

### Isolation procedure

For the first isolation, 25 g mature compost was added to 250 mL of CTVM-CMC in a 500 mL flask and shaken for 3 h at 150 rpm at 65 °C. After 3 h, compost was sieved with a 3 mm pore size filter and dilution series were plated on CTVMy-CMC and CTVM-CMC and grown aerobically for 72 h. Subsequently, single colonies were picked and transferred to fresh plates containing either CTVMy-CMC or CTVM-CMC (according to their isolation plate). Identical plates lacking CMC were used as negative control. Grown isolates were transferred twice to assay consistent growth and eliminate false-positives. These false-positives present colonies that possibly grow on the degradation products produced by surrounding colonies. By transferring colonies to fresh plates we were able to eliminate those from the selection. Remaining isolates were then grown anaerobically for 96 h to assay their potential of anaerobic growth. The remaining isolates were used to obtain pure cultures and afterwards stored in 15 % glycerol at −80 °C.

In the second isolation, 10 g active compost was inoculated into 100 mL of CTVM in a 500 mL beaker and stirred for 5 min at room temperature. Due to the active state of the compost, oxygen levels are believed to be limited at the sampling site. Therefore, the isolation of microorganisms from this sample was initiated under anaerobic conditions to mimic the site of sampling. Dilution series were plated on CTVMy-CMC and CTVM-CMC and grown anaerobically for 72 h. Subsequently, single colonies were picked and each colony was streaked to plates containing CTVMy or CTVM (according to their isolation plate) with or without CMC and grown anaerobically at 65 °C for 168 h (7 days). Isolates lacking growth on the negative controls were transferred twice more to CTVM(y)-CMC plates to assay consistent growth and eliminate false-positives. Pure cultures were obtained on CTVMy-glc plates and subsequently stored in 15 % glycerol stocks at −80 °C.

## 16S rRNA-encoding gene identification and phylogenetic analysis

Single colonies were picked from plates and inoculated to pre-warmed CTVMy medium with 1 % w/v glucose. When cultures reached the exponential phase (OD_600_ between 0.5 and 1) 2 µL was transferred to a PCR tube. PCR mix was added containing 1.25 units of DreamTaq DNA polymerase (Fermentas), 10× DreamTaq buffer (Fermentas), 100 µM of dNTPs (Fermentas), 0.2 µM of both primers GM3 (AGAGTTTGATCATGGC) and GM4 (TACCTTGTTACGACTT) and milliQ water in a total volume of 50 µL [21]. PCR products were checked on 1 % agarose gels and products were purified using a GeneJet PCR purification kit (Fermentas). CloneManager software was used to assemble and manually curate the GM3 and GM4 sequences into one contig after which BLASTn was used for identification against the non-redundant nucleotide collection of the NCBI database. Contigs were aligned using Mega6 [22] software and MUSCLE v3.8.31 [23] with 5 iterations and, subsequently, were trimmed to equal length using Jalview version 2.0 [24]. The 94 sequences have been submitted to NCBI under GenBank accession numbers KX113522–KX113615. A phylogenetic tree was created using the neighbor-joining method [25] and a bootstrap analysis [26] was done using 1000 replicates. Type strains used in the phylogenetic tree were derived from the following GenBank accession numbers: NR_043021.1 (*G. thermodenitrificans* DSM 465), NR_043022.1 (*G. thermoglucosidasius* DSM 2542), NR_028708.1 (*G. caldoxylosilyticus* DSM 12041), NR_115285.1 (*G. kaustophilus* DSM 7263), FN428684.1 (*G. thermoleovorans* DSM 5366), NZ_CP010052.1 (*B. subtilis* DSM 402).

### Selection

All pure strains were inoculated from glycerol stocks to plates containing CTVMy-CMC and CTVMy-xylan and subsequently incubated anaerobically for 48 h. Plates were then washed using milliQ water to remove all cells and subsequently stained for 5 min using a 0.1 % (w/w) Congo red dye. After staining, plates were de-stained for 15 min using 1 M NaCl under constant swirling. Clearing zones were identified visually and graded using a 4 step scale ranging from 0 to 4 with zero being no visible clearing zone and four representing the biggest clearing zone.

Screening on fermentation products was carried out in duplicate in 15 mL Greiner tubes with a total volume of 10 mL containing CTVMy medium at an initial pH of 6.5 with cellobiose or xylose as carbon substrate. Tubes were inoculated from plate colonies and incubated at 65 °C for 48 h with agitation at 50 rpm. After 48 h samples were taken for HPLC analysis on fermentation product profiles and quantities. Strains were then ranked based on highest total amount of products and highest total lactic acid formed. The average score of both cellobiose and xylose duplicates were combined and best scoring strains were then compared to the selected isolates from the Congo red plate assay (data not shown). Isolates with high enzymatic activity for either CMC and/or xylan combined with an above average ranking in the fermentation assay were selected to examine their genetic accessibility.

### Genetic accessibility and heterologous expression of the *pheB* reporter gene

Three isolates closely related to *G. thermodenitrificans* (99 %), *G. caldoxylosilyticus* (100 %) and *G. thermoleovorans* (99 %) were selected to evaluate genetic accessibility by electroporation. Strains were subjected to transformation in triplicate according to the protocols for *Geobacillus* described previously [14, 27] using the pNW33n plasmid isolated from *E. coli* dH5α. After 2 h recovery at 60 °C, electroporated cells were spread on LB2 plates containing 7 µg/mL of chloramphenicol and incubated for 24 h at 60 °C. Colonies that appeared within 48 h were subjected to PCR as described above to verify the presence of plasmid pNW33n inside the cells.

Optimisation of the transformation protocol was performed on strain T12. Three concentrations (0, 0.25 and 2.5 g/L) of K_2_HPO_4_ in the growth medium were used and washing of the cells was done 2 times with 50 mL of milliQ water followed by 2 washing steps, of 25 and 10 mL, respectively, using 10 % (v/v) glycerol [28].

As a demonstration of genetic accessibility, we cloned the reporter gene *pheB,* derived from *G. stearothermophilus* DSM 6285 (GenBank accession no. DQ146476.2) into strain T12. The *pheB* gene was synthesised by GeneArt (Thermo Scientific) as an expression construct using the constitutive promoter P*upp*T12. Promoter P*upp*T12 is derived from the upstream region (100nt) of the uracil phosphoribosyltransferase gene from T12 (Additional file 1: Figure S1). The promoter together with the *pheB* reporter gene were cloned to pNW33n between the Acc65I and PstI restriction sites. Activity of the reporter gene was tested by spraying colonies with 100 mM catechol followed by incubation at 55 °C for 5 min.

### Fermentations

Fermentations were performed in an Eppendorf DASGIP parallel bioreactor system or an Applikon fermentor system. In the Eppendorf DASGIP parallel bioreactor system glass reactors of 1.4 L and a working volume of 0.5 L was used with Dasgip control 4.0 to control the process. Glass reactors of 2 L and a working volume of 1 L were used in the Applikon fermentor system with BioXpert V2 for control. The conditions were as follows: Temperature was controlled at 65 °C, pH at 7.0 by addition of 3 M KOH and the stirring speed was 150 rpm. Antifoam 204 (Sigma-Aldrich) was added as required.

Pre-cultures were grown overnight from glycerol stock in 10 mL medium in a 50 mL tube at 65 °C and 150 rpm. The next morning 3 mL was transferred to 50 mL of medium in a 250 mL baffled Erlenmeyer and grown for 2 h before 2 % (v/v) was inoculated to the reactor. Samples of 2 mL were taken for off-line OD-measurements by determining the turbidity at 600 nm. Concentrations of sugar and fermentation products were determined by HPLC.

### HPLC

Sugars and fermentation products were measured using an ICS5000 HPLC system from Thermo Scientific equipped with a Dionex DP pump, Dionex AS-AP autosampler, Dionex VWD UV detector operated at 210 nm and Shodex RI detector at 35 °C. An Aminex HPX-87H cation-exchange column was used with a mobile phase of 8 mM H_2_SO_4_ and was operated at 0.8 mL/min and 60 °C. All samples were diluted with 10 mM dimethyl sulfoxide in 4 mM H_2_SO_4_ in a ratio of 5:1 sample/internal standard.

## Results

To isolate facultative anaerobic thermophiles capable of degrading cellulose and/or xylan, mature and active compost of plant materials were sampled with temperatures of 35 and 65 °C, respectively, at time of isolation.

### Isolation

The mature compost was suspended in CTVM medium at 65 °C and dilution series were plated on CTVM-CMC with or without 0.5 g/L yeast extract at 65 °C to select for thermophiles. Addition of yeast extract resulted in approximately 1.5 times more colonies. From both media, 130 isolates were picked and grown aerobically for almost 4 days. Clear differences in growth speed/lag time and recovery rate were observed in favour of plates containing yeast extract (Additional file 2: Table S1). The control plates without carbon source (CTVM and CTVMy) showed no or very little growth of the isolates, indicating that CMC was required to obtain the growth monitored. Growth of some isolates became visible later than that of others, which may indicate either slower growth rates or an elongated lag phase, due to growth on degradation products of other isolates. When isolates were streaked again to fresh medium, lag time was shortened and the amount of recovered isolates decreased, possibly due to the elimination of false-positives (Additional file 2: Table S1).

The active compost sample used in the second isolation was resuspended in CTVM medium, plated on CTVMy-CMC medium and incubated anaerobically at 65 °C for 72 h. After incubation, 130 colonies from the CTVMy-CMC plates and 130 colonies from CTVM-CMC plates were isolated and subsequently transferred to fresh plates. None of the isolates from the CTVM-CMC plates were recovered and only 17 could be recovered from the CTVMy-CMC plates. Further transfers demonstrated that maintaining the isolates on CMC medium was often unsuccessful and revival from glycerol stocks was even more challenging (Table 1).

**Table 1:** Isolate numbers recovered after multiple transfers on both CTVM-CMC and CTVMy-CMC media

Isolation round	Media	Isolates	Recovery 1st transfer	Recovery 2nd transfer	Recovery 3th transfer	Recovery −80 stock
1st (aerobic)	CTVM-CMC	130	63	31	31	9
	CTVMy-CMC	130	99	79	79	79
2nd (anaerobic)	CTVM-CMC	130	0	0	0	0
	CTVMy-CMC	130	17	16	13	6
Total		520	179	126	123	94

From the initial 520 isolates 94 have been recovered

### Selection

**Fig. 1:**
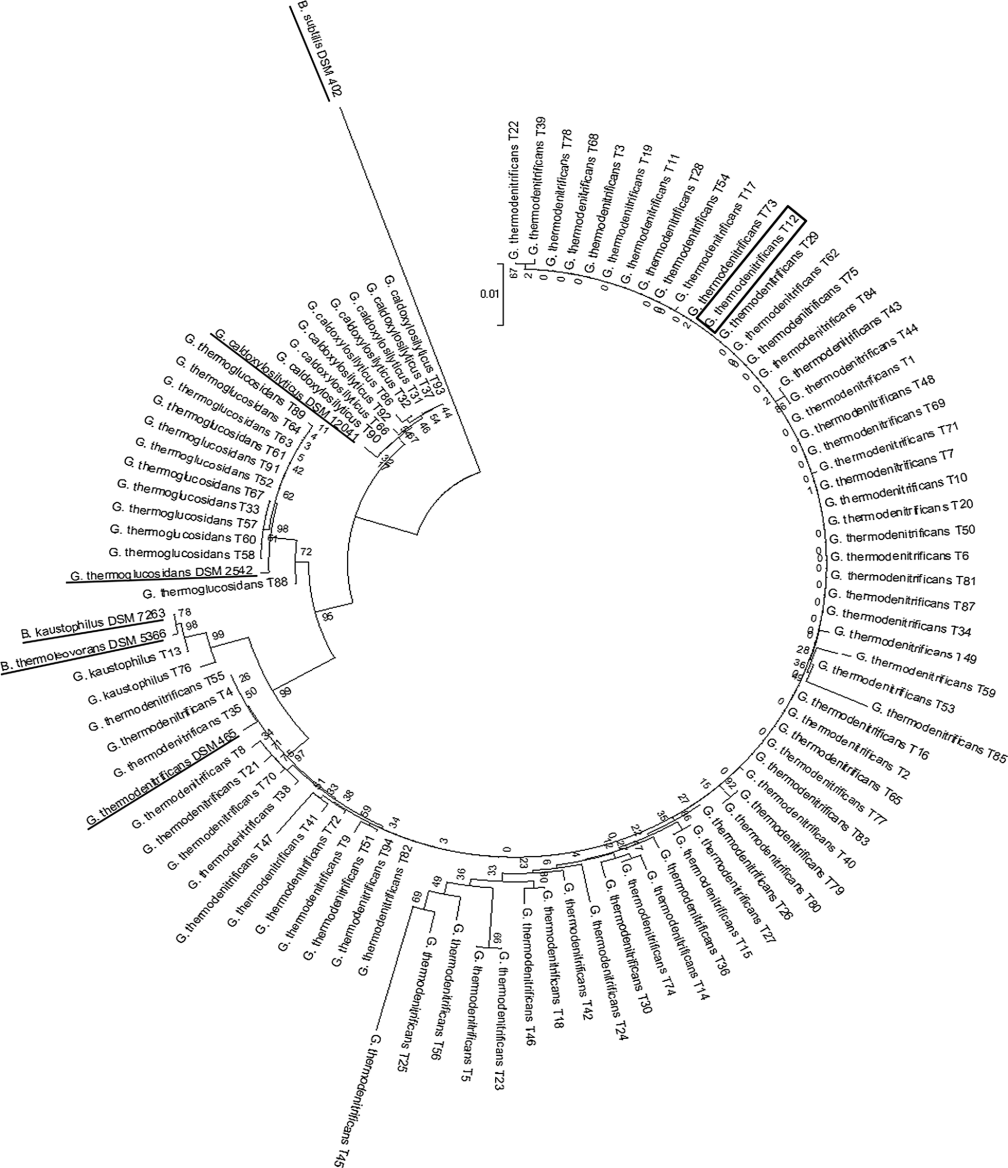
Phylogenetic tree of 16S rRNA gene sequences of *Geobacillus* isolates and their type strains (*underlined*). Phylogenetic tree was constructed by the Neighbor-joining method and tested with the bootstrap method using 1000 replicates. Selected isolate *G. thermodenitrificans* T12 is indicated by a *black box*

Pure cultures obtained from both isolation rounds were subjected to 16S rRNA-encoding gene sequencing for identification. All 94 isolates belonged to the *Geobacillus* genus (Fig. 1). The majority, 73 of 94 isolates, were *G. thermodenitrificans*. The remainder was classified as *G. thermoglucosidans* (11), *G. caldoxylosilyticus* (8) and *G. kaustophilus* (2).

Further selection was based on fermentation product profiles. Isolates were pre-grown on CTVMy-glc and subsequently transferred to fresh CTVMy media containing either glucose or xylose as the carbon source. After 48 h fermentation products were measured by HPLC (Additional files 3, 4: Tables S2, S3). All strains produced lactic acid as their main fermentation product. Besides lactic acid, acetic acid was present in all cultures. In some cases, independent of the amount of lactic acid, ethanol and formate were also produced. Succinate production was seen in some cultures, with *G. thermodenitrificans* T78 showing the highest average concentration of 4.3 mM succinic acid accounting for 19.3 % (mol/mol) of the total products (data not shown). All strains belonging to *G. thermoglucosidans* showed similar production profiles implying low variability within this genus in comparison to for instance *G. caldoxylosilyticus*. *G. thermoglucosidans* is highly represented in the 25 strains with the highest organic acid production titers. This is demonstrated by the presence of 10 out of 11 strains in the cellobiose fermentations ranking and 8 of 11 strains in the xylose fermentations ranking (Additional files 3, 4; Tables S2, S3). Contrary to this observation, *G. thermoglucosidans* is underrepresented in the ranking of the 25 best lactic acid producers with only 3 (C6) and 4 (C5) strains listed. Under such conditions *G. thermodenitrificans* has a more homolactic phenotype while *G. thermoglucosidans* has a more mixed acid fermentation phenotype (data not shown). The mixed acid fermentation profiles of *G. thermoglucosidans* are in line with a previous study that demonstrated a mixed acid fermentation profile under micro-aerobic conditions [29].

The list of strains producing the highest amounts of lactic acid from cellobiose fermentation was overlaid with the list of isolates producing most lactic acid on xylose. No isolates belonging to *G. caldoxylosilyticus* or *G. kaustophilus* appeared in both lists. In addition to the fermentation assays described above, all 94 isolates were subjected to Congo red stain after growth on CMC and beech wood xylan. About 24 strains degraded CMC, as indicated by halo formation after staining with the Congo red dye. However, after repeated assays using Congo red it became evident that the formation of halos was highly irreproducible, although growth was similar in most cases. When grown on xylan, halos were more pronounced and a clear degradation was observed for about half of the isolates. Strain T12 showed strong and reproducible breakdown of xylan. Based on the concentrations of the products in the cellobiose and xylose fermentations, and the reproducibility of the plate assays, strains T12, T62, and T85 (all originating from the 1st isolation round) were considered the best isolates for further study.

### Electroporation and optimisation of transformation

The genetic accessibility of these three selected isolates were then evaluated by electroporation performed based on protocols described previously [14, 30], with some modifications. Cells were transformed by electroporation using the *E. coli*–*Bacillus* shuttle vector pNW33n, a vector that has been widely used in thermophilic bacilli [17, 31, 32]. Colonies found to be chloramphenicol-resistant were streaked to fresh LB2 plates supplemented with chloramphenicol to eliminate false-positives and successful transformation was confirmed by colony PCR. Following this, plasmids were isolated from the positive clones and subjected to restriction analysis to confirm integrity, before transfer back to *E. coli.* While T62 and T85 did not yield any colonies, transformation of strain T12 reproducibly did. In all cases the colonies obtained for T12 were positive for plasmid uptake. Although the efficiency was low, the high reproducibility provided the opportunity for optimisation. Different culture media and wash protocols were evaluated (Table 2), and a combination of reduced concentrations of K_2_HPO_4_ with a wash protocol derived from [28], proved to be the best protocol with an optimum CFU/µg DNA of 1.7 × 10^4^. The LB2 medium initially used resulted in a short pulse time constant of 3.7 ms. However, changing this growth medium to a variant with a lower salt concentration resulted in an increased pulse time constant and a CFU/µg DNA of 1240, which was in line with the results seen in previous research [17]. Consequently, decreasing the salt concentrations in the growth medium LB2 resulted in the cell pellets becoming less dense, leading to loss of many cells during washing. To prevent the loss of cells, 0.25 g/L K_2_HPO_4_ was added and wash buffers were changed to milliQ water and 10 % glycerol, to increase the osmotic shock that results in weakening of the bacterial cell wall. Several variables need to be balanced to obtain a successful electroporation protocol. For example with a more severe wash protocol, the cells will be weaker during electroporation, which will probably require a lower voltage to reduce further damage to the cells. Although no statistical underpinning was performed, our protocol is demonstrated to be reproducible.

**Table 2:** Overview of transformation optimisations for *G. thermodenitrificans* T12

Parameter changed	Medium	Growth^a^ (h)	Final OD^b^	Voltage	Ω	µF	Cuvette width (mm)	Ptd^c^ (ms)	DNA^d^ (µg)	CFU/ µg DNA
N.A.	LB2 (2.50 g/L K_2_HPO_4_)	2	1.01	2000	200	25	2	3.7	3.031	4
Medium	LB2 (0.25 g/L K_2_HPO_4_)	1.67	0.96	2000	200	25	2	5.1	1.613	1240
Medium	LB2 (0.00 g/L K_2_HPO_4_)	1.66	0.96	2000	200	25	2	5	1.613	22
Wash buffer^e^	LB2 (2.50 g/L K_2_HPO_4_)	1.67	0.96	2000	200	25	2	5.2	1.613	19
Medium/wash buffer^e^	LB2 (0.00 g/L K_2_HPO_4_)	1.66	0.96	2000	200	25	2	5.4	1.613	2988
Medium/wash buffer^e^	LB2 (0.25 g/L K_2_HPO_4_)	1.83	0.96	2000	200	25	2	5.5	1.564	17,071

^a^ Time of growth after dilution of the overnight pre-culture

^b^ Final OD_600_ of the culture after growth prior to the washing step

^c^ Pulse time duration in milliseconds

^d^ Amount of plasmid (pNW33n) DNA added for transformation

^e^ Wash protocol was changed to 2 times washing with milliQ water (50 mL) followed by washing with 25 and 10 mL milliQ water +10 % glycerol

To verify the potential of the T12 genetic accessibility, we introduced *pheB* as a reporter gene. The reporter gene *pheB*, derived from *G. stearothermophilus* DSM 6285 (GenBank accession no. DQ146476.2), encodes a heterologous catechol 2,3-dioxygenase (C23O). The *pheB* gene is demonstrated to function as a reporter gene in *G. thermoglucosidans*, where it has been used for the quantification of promoter strength [33]. The protein C23O oxidises catechol to form 2-hydroxymuconic semialdehyde (2-HMSA) which has a bright yellow colour. Colonies harbouring the *pheB* expression construct can, therefore, be visualised by spraying them with 100 mM catechol and incubating them for 5 min at 55 °C. A bright yellow colour was observed on all colonies harbouring the *pheB* expression construct, while no colour change was observed in colonies harbouring empty pNW33n plasmids (Additional file 5: Figure S2).

### Growth and fermentation of T12

Fermentation characteristics of *G. thermodenitrificans* T12 were evaluated under micro-aerobic conditions. To determine an optimal pH, multiple pH-controlled fermentations were carried out in MMy media containing 30 g/L glucose, with a pH range of 5.5–8.5. Both growth and product formation were monitored during the fermentation (data not shown) to determine the optimal conditions. Growth was observed between pH 5.5 and 7.5, with an optimum between pH 6.5 and 7.5. This optimal growth pH of 7.5 coincided with the highest amount of lactic acid production followed closely by the culture at pH 6.5.

**Fig. 2:**
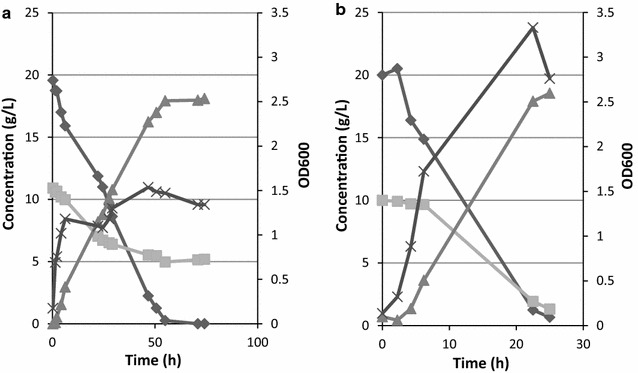
Fermentations of *G. thermodenitrificans* T12 on a 2:1 glucose/xylose mixture. Fermentations were carried out at pH 7.0 at 150 rpm. Fermentations started with a 0.5 L air headspace without additional sparging and media consisted of 500 mL MMy (**a**) or MMy+ (**b**).  glucose;  xylose;  lactic acid; × OD_600_

To assay the potential of strain T12 in converting biomass derived sugars, its fermentation products and productivity thereof, were quantified in fermentations with glucose, xylose and mixtures of these sugars as carbon source. For all carbon substrates, both MMy media (containing 0.5 g/L yeast extract and NH_4_NO_3_) as well as MMy+ media (containing 5 g/L yeast extract and NH_4_Cl) were evaluated (Fig. 2; Table 3). Ammonium nitrate was added to MMy medium to stimulate biomass formation [34].

**Table 3:** Overview of fermentations of *G. thermodenitrificans* T12 on glucose, xylose and glucose/xylose mixtures

Media	Substrate	Time (h)	Cons. (g)	Cons. rate (g/L/h)	Lactic acid production (g)	Lactic acid productivity	Lactic acid yield	Lactic acid yield
						(g/L/h)	(g product/g substrate consumed)	(% of theoretical yield)
MMy	30 g/L glucose	25.2	9.43	0.37	3.10	0.12	0.33	10.3
MMy	30 g/L xylose	25	6.93	0.28	3.83	0.15	0.55	12.8
MMy	20 g/L glucose + 10 g/L xylose	54.95	19.87 glucose 6.55 xylose	0.33 glucose 0.11 xylose	17.93	0.30	0.69	59.8
MMy+	30 g/L glucose	27	11.35	0.41	8.48	0.30	0.74	28.3
MMy+	30 g/L xylose	27	15.23	0.54	8.73	0.31	0.57	29.1
MMy+	20 g/L glucose + 10 g/L xylose	25	14.51 glucose 7.05 xylose	0.50 glucose 0.24 xylose	20.27	0.71	0.94	67.6

Fermentations were carried out at pH 7.0 at 150 rpm. Fermentations started with a 0.5 L air headspace without additional sparging. MMy media contained 0.5 g/L of yeast extract, whereas MMy+ media contained 5 g/L of yeast extract

The average lactic acid productivity on 30 g/L glucose or 30 g/L xylose, either on minimal or rich medium, did not differ significantly. However, productivities differ when comparing a single carbon source on minimal and rich medium (Table 3). Co-fermentation of both glucose and xylose was performed in both media (Fig. 2; Table 3). When MMy medium with a 2:1 ratio of glucose:xylose was used, a total of 17.9 g lactic acid was produced in 55 h with an overall yield of 0.7 g lactic acid/ g sugar consumed. From the start of the fermentation, both glucose and xylose were consumed simultaneously though the consumption rates for xylose were lower in comparison to glucose (Table 3). Fermentations in MMy+ medium showed improved consumption rates, productivity and yield of lactic acid produced per amount of consumed substrate.

In all mixed sugar fermentations the xylose consumption rate was 2–3 times slower when compared to the consumption rate of glucose. To exclude the possibility that the sugar concentration is instrumental in this observation, a glucose/xylose co-fermentation was completed with equal concentrations of the two sugars (10 g/L) in MMy medium. Observed consumption rates of 0.2 g/L/h for glucose and 0.1 g/L/h for xylose confirm that glucose is in fact favoured over xylose in co-fermentation.

**Fig. 3:**
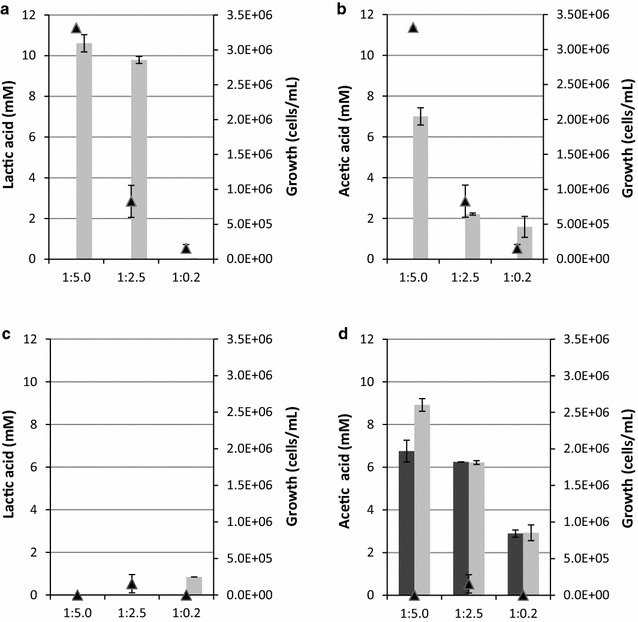
Xylan fermentations by *G. thermodenitrificans* T12. Lactic acid production (**a**, **c**) and acetic acid production (**b**, **d**) of strain T12 on MMy (**a**, **b**) and MMy+ (**c**, **d**) media with xylan (*light-grey bars*) and without xylan (*dark-grey bars*). The cultures were grown in 50 mL Greiner tubes at pH 6.5 and 150 rpm. Ratios on the* X-axis* represent the ratio of medium and headspace during fermentation. Growth (▴) is expressed as increase of cells on xylan compared to cultures without xylan

The Congo Red assay showed that strain T12 was capable of degrading beech wood xylan on solid medium. To quantify its fermentation products liquid cultures with 10 g/L beech wood xylan as carbon source were used. Fermentations were carried out in 50 mL Greiner tubes with different volumes of head space and 250 mL shake flasks to demonstrate the impact of oxygen (Fig. 3). Under micro-aerobic conditions in low nutrient (MMy) medium (Fig. 3a, b), lactic acid (11 mM) was the main fermentation product followed by acetic acid. In 250 mL baffled Erlenmeyer shake flasks using 50 mL culture (1:5 with more aeration), lactic acid concentration was reduced to 0.55 (±0.04) mM and acetic acid increased to 19 (±0.04) mM (data not shown). Use of the rich (MMy+) medium under any condition resulted in minimal amounts of lactic acid production (Fig. 3c) and increased acetate production, due to the conversion of the yeast extract (Fig. 3d). In addition to the increased lactic acid production we noticed an increase in cell densities in minimal media with xylan in comparison to cultures on minimal media without xylan (Fig. 3a). In rich media no significant difference in cell densities were observed between cultures with and without xylan, likely due to high growth rates on yeast extract (Fig. 3c).

## Discussion

This study was aimed at the discovery of novel industrially relevant strains that harbour saccharolytic enzymes to degrade cellulose and xylan have lactic acid as their main fermentation product and are genetically accessible. We describe the isolation of 94 facultative anaerobic *Geobacillus* strains that were capable of fermentation of both C6 and C5 sugars under micro-aerobic and anaerobic conditions. For isolate *G. thermodenitrificans* T12 direct conversion of beech wood xylan to lactic acid was demonstrated. The selection methods yielded several isolates that were capable of degrading xylan and cellulose. All strains described in this study were isolated from compost at both moderate and high temperature by repetitive growth on both CMC and xylan. It has been previously shown that *Geobacillus* was the dominant genus in “mature” compost [35–37]. However, it cannot be concluded if the ratio of *Geobacillu*s species found in this study represents the composition of the *Geobacillus* community in the compost heap sampled, or results from selection by the sampling and isolation methods. In prior studies [17], compost heaps on the same site were sampled and screened for thermophilic organisms. The TMM media used in the study of Bosma et al. [17] contained 0.9 g NaNO_3_ which led, at 65 °C, to the isolation of mainly *G. thermodenitrificans* strains. When both vitamins and metals were added, a larger diversity in *Geobacillus* species was found. In isolations done at 60 °C without nitrate in the medium approximately half the isolates belonged to *Geobacillus* but no *G. thermodenitrificans* isolates were found (E F Bosma, unpublished data). With the selection criteria used in this study, it was anticipated that geobacilli would be isolated. The overrepresentation of *G. thermodenitrificans* (79 % of isolates) in this isolation procedure, however, was surprising since nitrate was excluded from the isolation media to prevent this species benefiting over other species.

All strains were isolated after repeated growth on CMC and beech wood xylan. Degradation of xylan was always detectable and most strains showed clearing zones when assayed on Congo red plates. In contrast, the results on CMC in Congo red assays appeared difficult to reproduce. Although never described for *Geobacillus* or closely related species, the irreproducibility is most likely caused by regulation of the expression of saccharolytic enzymes, as has been described for xylanolytic enzymes [38]. The latter study describes that the hemicellulose utilisation (HUS) locus is most likely induced by small amounts of free xylose. It is known that the coverage of the HUS locus varies substantially among *Geobacillus* species and even differs per strain [10]. From our study it has become clear that within geobacilli there is major variation in the regulation of the xylanolytic cassette. In apparently identical situations, the breakdown of xylan differs greatly between *Geobacillus* species and even between strains of the same species. Regulation of cellulase gene expression has not yet been described for *Geobacilli* and details of its control remain elusive. Growth is no direct evidence for the presence of extracellular cellulases although free glucose is limited in this substrate which implies at least some cellulolytic activity to be present.

When grown under micro-aerobic conditions, all isolates generated lactic acid as their main fermentation product from glucose and xylose. Acetate and succinate were always present as minor by-products, and occasionally, formate and ethanol were produced. Isolates belonging to the *G. thermoglucosidans* clade showed low differentiation in terms of fermentation products and amounts, whereas *G. caldoxylosilyticus and G. thermodenitrificans* produce quite a diverse spectrum.

Genetic accessibility was tested for three selected isolates. Strain T12 was found to be reproducibly transformable, albeit with low efficiencies of 3–5 transformants per µg of plasmid DNA. In previous research [17], only 2 of 25 strains of *G. thermodenitrificans* could be transformed. The transformation efficiencies of *G. thermodenitrificans* isolates in the latter study are in line with the efficiency of transformation described here. Efficient transformation of a *G. thermodenitrificans* strain by electroporation has been described before [16] yielding approximately 2.8 × 10^6^ colonies/µg DNA. However, this protocol resulted in only 3–5 T12 transformants/µg of plasmid DNA. The efficiency reported by Kananavičiute and Čitavičius [16] was obtained using plasmid DNA isolated from the same strain, circumventing possible degradation by a native restriction modification system. Conversely, there is no literature available describing a systematic approach in optimising electroporation protocols for *Geobacillus* species. We have optimised the transformation for *G. thermodenitrificans* T12 by adjusting the concentration of K_2_HPO_4_ in its growth media used in preparation of making the cells competent. The change of wash buffers further increased the transformation efficiency to CFU/µg DNA of 1.7 × 10^4^. With this transformation efficiency we were able to introduce the heterologous reporter gene *pheB* to T12. The expression of the *pheB* gene, controlled by a constitutive promoter (PuppT12), shows the potential of strain T12 for further genetic engineering. Strain T12 was selected based on its ability to grow on CMC plates, its reproducible xylan degradation, an above average product titre on C6 and C5 fermentations, and its genetic accessibility, which provides opportunities to develop this strain into a host for CBP processes. To reach this potential, the organism should be engineered to produce cellulolytic enzymes.

Strain T12 was found to have an optimum pH between 6.5 and 7.5, and temperature of 65 °C, identical to that of isolation. When T12 is grown on pure glucose or xylose the sugar consumption rate was almost equal, whereas during co-fermentation the glucose consumption rate was approximately two to three times as high compared to the xylose consumption rate. This indicates that the presence of glucose partially inhibits xylose uptake and/or metabolism. Reduced xylose consumption by *Geobacillus* in a glucose/xylose mixture has been reported before [29]. On average, hemicellulose makes up 25–35 % of lignocellulosic biomass, which makes simultaneous sugar uptake advantageous for the CBP economy [39]. There are many microbes capable of xylose consumption, however, most of these organisms suppress the uptake of xylose when glucose is present. This negative feedback results in sequential consumption of available sugars, leading to elongated fermentation times or accumulation of xylose in the reactor during fed-batch.

**Fig. 4:**
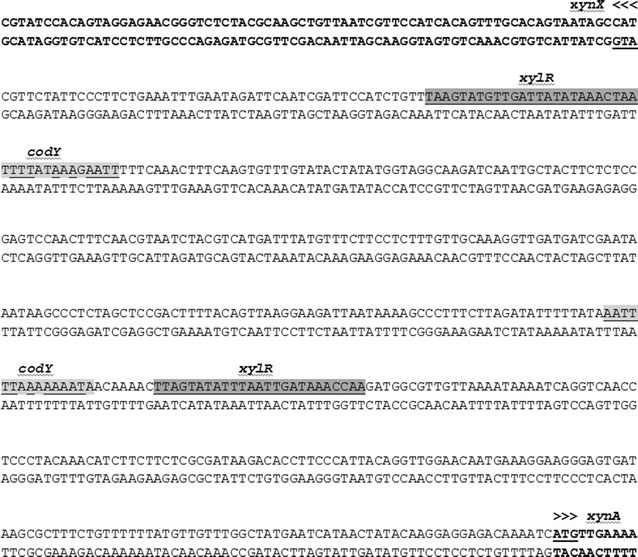
Intergenic region of the divergently oriented *xynX* and *xynA* genes. The predicted start codons of *xynA* and *xynX* genes are shown *boldface* and *underlined*. Predicted CodY-binding regions and predicted xylose regulator XylR-binding sites are *shaded grey*. Nucleotides matching the *B. subtilis* consensus sequences have been *underlined*. The CcpA-binding sites reported for *G. stearothermophilus* T6 [38] were not found

We have demonstrated the direct conversion of beech wood xylan into lactic acid by *G. thermodenitrificans* T12. The ratio of medium to headspace was important for this process, where increased oxygen transfer seems to positively affect xylan degradation, but negatively influences the ratio of lactic acid to acetic acid produced. Higher acetic acid production was seen with higher oxygen concentrations, while lactic acid was mostly produced in micro-aerobic conditions. The reduced degradation of xylan under high nutrient conditions (MMy+) was most likely caused by the repression of *xynA* by the global regulator CodY. It is known from *B. subtilis* that the CodY regulator is responsive to branched-chain amino acids (nutrient-rich conditions) [40]. A CodY orthologue has been found in the genome sequence of *G. thermodenitrificans* T12 (to be published), and two potential binding regions for CodY, based on the binding region consensus sequences from [38, 40], were identified in the promoter region of the T12 *xynA* gene (Fig. 4). For *G. stearothermophilus* T6, which has a xylanolytic operon similar to that of T12, CodY binding to the *xynA* promoter was demonstrated and its function as repressor of *xynA* expression was postulated [38]. Our results are in line with this hypothesis (Fig. 4c, d).

## Conclusions

In conclusion, a collection of 94 *Geobacillus* isolates were obtained from an initial selection of 520 thermophilic organisms, by screening for lactic acid production on both C6 and C5 sugars, together with the ability to degrade xylan and/or cellulose. Genetic accesibility was confirmed and optimised for one isolate, which was designated *G. thermodenitrificans* T12. This strain is able to grow on CMC plates, although Congo red assays are inconsistent. Strain T12 is capable of fermenting both xylose and glucose simultaneously with lactic acid as its main fermentation product. In addition, beechwood xylan was directly converted to lactic acid. The capacity of strain T12 to ferment xylan together with its genetic accessibility makes *G. thermodenitrificans* T12 a potential candidate for consolidated bioprocessing.

## Additional files

10.1186/s13068-016-0618-7 Sequence of the P*upp*T12 promoter including the first ATG codon of the uracil phosphoribosyltransferase gene.

10.1186/s13068-016-0618-7 Overview of the number of isolates recovered from the first isolation. Overview of the number of isolates recovered from the first isolation on CTVMy-CMC and CTVM-CMC media after 20 h, 48 h and 88 h. The pH was set at 6.5 and plates were incubated at 65 °C. Dilution series were plated from compost that had been shaken at 150 rpm for 3 h in the same medium and temperature.

10.1186/s13068-016-0618-7 HPLC data from isolates ranked on total organic acid production and total lactic acid production on cellobiose (C6).

10.1186/s13068-016-0618-7 HPLC data from isolates ranked on total organic acid production and total lactic acid production on xylose (C5).

10.1186/s13068-016-0618-7 *G. thermodenitrificans* T12 transformed with pNW33n (A1) and pNW33n+ *pheB* (B1). Colonies were sprayed with 100 mM catechol and incubated for 5 min at 55 °C. The colony containing pNW33n did not show any colour formation (A2) while the colony with the *pheB* gene under control of the constitutive uracil phosphoribosyltransferase promoter (P*upp*T12) shows a yellow colour (B2) indicating the conversion of catechol to 2-hydroxymuconic semialdehyde.

## Abbreviations

PLA: poly lactic acid
CBP: consolidated bioprocessing
HUS locus: hemicellulose utilisation locus
NCBI: National Centre For Biotechnology Information
CTVM: cellulolytic thermophile vitamin medium
MM: minimal medium
LB: luria bertani
CMC: carboxymethyl cellulose
Glc: glucose
OD: optical density
HPLC: high pressure liquid chromatography
O/N: overnight
RI: refractive index
CFU/µg DNA: colony forming units per microgram of plasmid DNA
Ptd: pulse time duration
