# Supplementary material for: Isolation of a genetically accessible thermophilic xylan degrading bacterium from compost
Source: Biotechnol Biofuels. 2016 Oct 6;9:210. doi: 10.1186/s13068-016-0618-7 (PMC5053077; doi:10.1186/s13068-016-0618-7)
Supplement: Supplementary file 1 — 10.1186/s13068-016-0618-7 Sequence of the PuppT12 promoter including the first ATG codon of the uracil phosphoribosyltransferase gene. [file 13068_2016_618_MOESM1_ESM.docx]

**Figure S1. Sequence of the PuppT12 promoter including the first ATG codon (in bolt) of the uracil phosphoribosyltransferase gene.**

5’ - TAAGTGTGCCTTTTCCTTTGCTTCAACGGTTGAACGGGCGCCCGTTTTCCAGTAGAATGT

ATAGAAGTGTACTGCATACATACGGAAGAGGAGATGACCT**ATG** - 3’
