# Supplementary material for: Isolation of a genetically accessible thermophilic xylan degrading bacterium from compost
Source: Biotechnol Biofuels. 2016 Oct 6;9:210. doi: 10.1186/s13068-016-0618-7 (PMC5053077; doi:10.1186/s13068-016-0618-7)
Supplement: Supplementary file 2 — 10.1186/s13068-016-0618-7 Overview of the number of isolates recovered from the first isolation. Overview of the number of isolates recovered from the first isolation on CTVMy-CMC and CTVM-CMC media after 20 h, 48 h and 88 h. The pH was set at 6.5 and plates were incubated at 65 °C. Dilution series were plated from compost that had been shaken at 150 rpm for 3 h in the same medium and temperature. [file 13068_2016_618_MOESM2_ESM.docx]

**TABLE S2. Overview of the number of isolates recovered from the first isolation.**

|  | 1st transfer | | | 2nd transfer | | |
| --- | --- | --- | --- | --- | --- | --- |
| Media | 20h | 48h | 88h | 20h | 48h | 88h |
| CTVMy-CMC | 28 | 94 | 99 | 62 | 79 | 79 |
| CTVM-CMC | 1 | 13 | 63 | 3 | 7 | 31 |

Overview of the number of isolates recovered from the first isolation on CTVMy-CMC and CTVM-CMC media after 20h, 48h and 88h. The pH was set at 6.5 and plates were incubated at 65°C. Dilution series were plated from compost that had been shaken at 150 rpm for 3h in the same medium and temperature.
