# Supplementary material for: Isolation of a genetically accessible thermophilic xylan degrading bacterium from compost
Source: Biotechnol Biofuels. 2016 Oct 6;9:210. doi: 10.1186/s13068-016-0618-7 (PMC5053077; doi:10.1186/s13068-016-0618-7)
Supplement: Supplementary file 3 — 10.1186/s13068-016-0618-7 HPLC data from isolates ranked on total organic acid production and total lactic acid production on cellobiose (C6). [file 13068_2016_618_MOESM3_ESM.docx]

**TABLE S3. HPLC data from isolates ranked on total organic acid production and total lactic acid production on cellobiose (C6)**

| Strain | Identification | Lactate **(mM)** | SD. | **Total (mM)** | SD. |  | Strain | Identification | **Lactate (mM)** | SD. | Total | SD. |
| --- | --- | --- | --- | --- | --- | --- | --- | --- | --- | --- | --- | --- |
| T62 | *G. thermodenitrificans* | 17.187 | 0.41 | **32.429** | 0.397 |  | T85 | *G. thermodenitrificans* | **19.727** | 0.017 | 27.906 | 0.216 |
| T87 | *G. thermodenitrificans* | 17.336 | 0.675 | **31.606** | 0.656 |  | T13 | *G. kaustophilus* | **17.772** | 0.334 | 22.334 | 0.342 |
| T83 | *G. thermodenitrificans* | 16.43 | 0.414 | **31.431** | 0.503 |  | T12 | *G. thermodenitrificans* | **17.669** | 0.191 | 28.885 | 0.309 |
| T39 | *G. thermodenitrificans* | 16.481 | 1.543 | **29.055** | 0.852 |  | T35 | *G. thermodenitrificans* | **17.414** | 0.666 | 21.168 | 0.646 |
| T12 | *G. thermodenitrificans* | 17.669 | 0.191 | **28.885** | 0.309 |  | T87 | *G. thermodenitrificans* | **17.336** | 0.675 | 31.606 | 0.656 |
| T85 | *G. thermodenitrificans* | 19.727 | 0.017 | **27.906** | 0.216 |  | T50 | *G. thermodenitrificans* | **17.277** | 1.156 | 20.471 | 1.463 |
| T64 | *G. thermoglucosidasius* | 14.652 | 0.182 | **27.212** | 0.587 |  | T40 | *G. thermodenitrificans* | **17.199** | 0.415 | 20.768 | 1.139 |
| T63 | *G. thermoglucosidasius* | 15.128 | 0.214 | **27.15** | 0.083 |  | T62 | *G. thermodenitrificans* | **17.187** | 0.41 | 32.429 | 0.397 |
| T57 | *G. thermoglucosidasius* | 15.053 | 0.528 | **27.102** | 0.358 |  | T48 | *G. thermodenitrificans* | **16.94** | 0.49 | 20.448 | 0.349 |
| T60 | *G. thermoglucosidasius* | 15.364 | 0.608 | **26.878** | 0.088 |  | T55 | *G. thermodenitrificans* | **16.87** | 0.991 | 23.051 | 0.713 |
| T61 | *G. thermoglucosidasius* | 15.065 | 0.173 | **26.805** | 0.136 |  | T45 | *G. thermodenitrificans* | **16.859** | 0.897 | 20.395 | 0.783 |
| T58 | *G. thermoglucosidasius* | 15.579 | 0.558 | **26.726** | 0.79 |  | T52 | *G. thermoglucosidasius* | **16.818** | 0.043 | 22.245 | 0.341 |
| T34 | *G. thermodenitrificans* | 14.815 | 0.432 | **26.687** | 0.495 |  | T44 | *G. thermodenitrificans* | **16.713** | 1.24 | 20.779 | 0.853 |
| T59 | *G. thermodenitrificans* | 15.514 | 0.132 | **26.551** | 1.476 |  | T49 | *G. thermodenitrificans* | **16.677** | 1.913 | 19.93 | 2.463 |
| T33 | *G. thermoglucosidasius* | 15.396 | 0.43 | **26.504** | 0.175 |  | T39 | *G. thermodenitrificans* | **16.481** | 1.543 | 29.055 | 0.852 |
| T93 | *G. caldoxylosilyticus* | 15.979 | 1.352 | **26.278** | 0.032 |  | T83 | *G. thermodenitrificans* | **16.43** | 0.414 | 31.431 | 0.503 |
| T88 | *G. thermoglucosidasius* | 15.178 | 0.542 | **23.068** | 0.336 |  | T26 | *G. thermodenitrificans* | **16.063** | 1.083 | 19.792 | 1.047 |
| T55 | *G. thermodenitrificans* | 16.87 | 0.991 | **23.051** | 0.713 |  | T93 | *G. caldoxylosilyticus* | **15.979** | 1.352 | 26.278 | 0.032 |
| T13 | *G. kaustophilus* | 17.772 | 0.334 | **22.334** | 0.342 |  | T51 | *G. thermodenitrificans* | **15.767** | 1.008 | 21.194 | 1.484 |
| T52 | *G. thermoglucosidasius* | 16.818 | 0.043 | **22.245** | 0.341 |  | T30 | *G. thermodenitrificans* | **15.725** | 0.577 | 19.35 | 0.385 |
| T31 | *G. caldoxylosilyticus* | 13.625 | 0.698 | **21.616** | 0.262 |  | T91 | *G. thermoglucosidasius* | **15.604** | 0.023 | 20.773 | 0.015 |
| T73 | *G. thermodenitrificans* | 15.435 | 0.286 | **21.415** | 0.45 |  | T58 | *G. themoglucosidasius* | **15.579** | 0.558 | 26.726 | 0.79 |
| T51 | *G. thermodenitrificans* | 15.767 | 1.008 | **21.194** | 1.484 |  | T42 | *G. thermodenitrificans* | **15.538** | 3.213 | 20.06 | 1.884 |
| T35 | *G. thermodenitrificans* | 17.414 | 0.666 | **21.168** | 0.646 |  | T59 | *G. thermodenitrificans* | **15.514** | 0.132 | 26.551 | 1.476 |
| T54 | *G. thermodenitrificans* | 15.371 | 0.284 | **21.063** | 0.74 |  | T73 | *G. thermodenitrificans* | **15.435** | 0.286 | 21.415 | 0.45 |
