# Supplementary material for: Isolation of a genetically accessible thermophilic xylan degrading bacterium from compost
Source: Biotechnol Biofuels. 2016 Oct 6;9:210. doi: 10.1186/s13068-016-0618-7 (PMC5053077; doi:10.1186/s13068-016-0618-7)
Supplement: Supplementary file 4 — 10.1186/s13068-016-0618-7 HPLC data from isolates ranked on total organic acid production and total lactic acid production on xylose (C5). [file 13068_2016_618_MOESM4_ESM.docx]

**TABLE S4. HPLC data from isolates ranked on total organic acid production and total lactic acid production on xylose (C5)**

| Strain | Identification | Lactate **(mM)** | SD. | **Total (mM)** | SD. |  | Strain | Identification | **Lactate (mM)** | SD. | Total **(mM)** | SD. |
| --- | --- | --- | --- | --- | --- | --- | --- | --- | --- | --- | --- | --- |
| T58 | *G. thermoglucosidasius* | 13.225 | 1.447 | **27.555** | 0.272 |  | T90 | *G. caldoxylosilyticus* | **18.553** | 0.264 | 23.964 | 1.191 |
| T60 | *G. thermoglucosidasius* | 15.638 | 0.054 | **27.513** | 0.187 |  | T40 | *G. thermodenitrificans* | **18.021** | 0.198 | 22.553 | 0.074 |
| T61 | *G. thermoglucosidasius* | 14.207 | 0.456 | **27.446** | 0.121 |  | T1 | *G. thermodenitrificans* | **17.681** | 0.097 | 22.225 | 0 |
| T57 | *G. thermoglucosidasius* | 14.331 | 0.746 | **27.414** | 0.689 |  | T6 | *G. thermodenitrificans* | **17.549** | 0.355 | 23.335 | 1.925 |
| T63 | *G. thermoglucosidasius* | 15.583 | 0.669 | **27.322** | 0.295 |  | T70 | *G. thermodenitrificans* | **17.501** | 0.002 | 21.148 | 0.182 |
| T59 | *G. thermodenitrificans* | 11.428 | 0.692 | **26.955** | 0.999 |  | T88 | *G. thermoglucosidasius* | **17.422** | 0.057 | 21.555 | 0.198 |
| T85 | *G. thermodenitrificans* | 14.14 | 0.122 | **25.709** | 1.628 |  | T62 | *G. thermodenitrificans* | **17.366** | 0.209 | 24.157 | 2.753 |
| T89 | *G. thermoglucosidasius* | 16.112 | 0.823 | **25.705** | 0.074 |  | T80 | *G. thermodenitrificans* | **17.36** | 0.177 | 21.029 | 0.137 |
| T39 | *G. thermodenitrificans* | 11.184 | 0.104 | **25.596** | 1.925 |  | T7 | *G. thermodenitrificans* | **17.311** | 0.663 | 23.451 | 0.772 |
| T51 | *G. thermodenitrificans* | 16.242 | 1.527 | **25.353** | 1.386 |  | T44 | *G. thermodenitrificans* | **17.165** | 0.314 | 21.232 | 0.75 |
| T91 | *G. thermoglucosidasius* | 16.489 | 0.167 | **25.194** | 0.639 |  | T92 | *G. caldoxylosilyticus* | **17.164** | 0.158 | 25.131 | 0.903 |
| T92 | *G. caldoxylosilyticus* | 17.164 | 0.158 | **25.131** | 0.903 |  | T52 | *G. thermoglucosidasius* | **16.916** | 0.47 | 24.922 | 0.556 |
| T52 | *G. thermoglucosidasius* | 16.916 | 0.47 | **24.922** | 0.556 |  | T71 | *G. thermodenitrificans* | **16.671** | 0.154 | 20.371 | 0.047 |
| T86 | *G. caldoxylosilyticus* | 15.527 | 0.619 | **24.433** | 0.141 |  | T4 | *G. thermodenitrificans* | **16.556** | 0.45 | 19.97 | 0.139 |
| T62 | *G. thermodenitrificans* | 17.366 | 0.209 | **24.157** | 2.753 |  | T91 | *G. thermoglucosidasius* | **16.489** | 0.167 | 25.194 | 0.639 |
| T24 | *G. thermodenitrificans* | 15.131 | 0.26 | **24.107** | 0.004 |  | T25 | *G. thermodenitrificans* | **16.415** | 2.247 | 20.405 | 2.194 |
| T90 | *G. caldoxylosilyticus* | 18.553 | 0.264 | **23.964** | 1.191 |  | T51 | *G. thermodenitrificans* | **16.242** | 1.527 | 25.353 | 1.386 |
| T7 | *G. thermodenitrificans* | 17.311 | 0.663 | **23.451** | 0.772 |  | T2 | *G. thermodenitrificans* | **16.151** | 0.094 | 20.854 | 0.291 |
| T6 | *G. thermodenitrificans* | 17.549 | 0.355 | **23.335** | 1.925 |  | T89 | *G. thermoglucosidasius* | **16.112** | 0.823 | 25.705 | 0.074 |
| T40 | *G. thermodenitrificans* | 18.021 | 0.198 | **22.553** | 0.074 |  | T60 | *G. thermoglucosidasius* | **15.638** | 0.054 | 27.513 | 0.187 |
| T10 | *G. thermodenitrificans* | 13.88 | 0.759 | **22.482** | 0.404 |  | T63 | *G. thermoglucosidasius* | **15.583** | 0.669 | 27.322 | 0.295 |
| T1 | *G. thermodenitrificans* | 17.681 | 0.097 | **22.225** | 0 |  | T26 | *G. thermodenitrificans* | **15.549** | 0.958 | 18.806 | 1.41 |
| T78 | *G. thermodenitrificans* | 14.062 | 0.105 | **22.078** | 0.124 |  | T86 | *G. caldoxylosilyticus* | **15.527** | 0.619 | 24.433 | 0.141 |
| T11 | *G. thermodenitrificans* | 11.909 | 0.244 | **21.907** | 5.901 |  | T31 | *G. caldoxylosilyticus* | **15.514** | 1.405 | 21.728 | 0.426 |
| T31 | *G. caldoxylosilyticus* | 15.514 | 1.405 | **21.728** | 0.426 |  | T15 | *G. thermodenitrificans* | **15.317** | 0.474 | 19.554 | 0.741 |
