# Supplementary figures and images for: Isolation of a genetically accessible thermophilic xylan degrading bacterium from compost
Source: Biotechnol Biofuels. 2016 Oct 6;9:210. doi: 10.1186/s13068-016-0618-7 (PMC5053077; doi:10.1186/s13068-016-0618-7)

**Figure S5.**

**B**

**A**


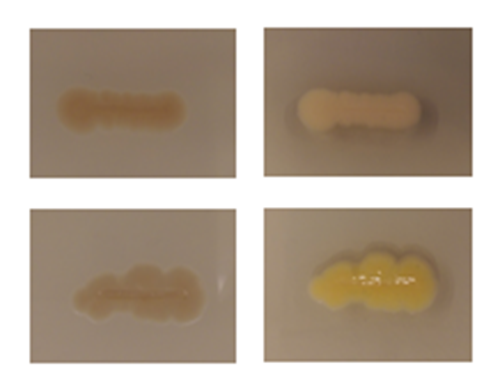


B1

B2

A2

A1

Supplement: Supplementary file 5 — 10.1186/s13068-016-0618-7 G. thermodenitrificans T12 transformed with pNW33n (A1) and pNW33n+ pheB (B1). Colonies were sprayed with 100 mM catechol and incubated for 5 min at 55 °C. The colony containing pNW33n did not show any colour formation (A2) while the colony with the pheB gene under control of the constitutive uracil phosphoribosyltransferase promoter (PuppT12) shows a yellow colour (B2) indicating the conversion of catechol to 2-hydroxymuconic semialdehyde. [file 13068_2016_618_MOESM5_ESM.docx]
